# Analgesic efficacy of intrathecal morphine and bupivacaine during the early postoperative period in patients who underwent robotic-assisted laparoscopic prostatectomy: a prospective randomized controlled study

**DOI:** 10.1186/s12894-021-00798-4

**Published:** 2021-02-26

**Authors:** Jung-Woo Shim, Yun Jeong Cho, Hyong Woo Moon, Jaesik Park, Hyung Mook Lee, Yong-Suk Kim, Young Eun Moon, Sang Hyun Hong, Min Suk Chae

**Affiliations:** 1 Department of Anesthesiology and Pain Medicine, Seoul St. Mary’s Hospital, College of Medicine, The Catholic University of Korea, 222, Banpo-daero, Seocho-gu, Seoul, 06591 Republic of Korea; 2 Department of Urology, Seoul St. Mary’s Hospital, College of Medicine, The Catholic University of Korea, Seoul, Republic of Korea

**Keywords:** Intrathecal, Morphine, Bupivacaine, Robotic-assisted laparoscopic surgery

## Abstract

**Background:**

The present study was performed to investigate the analgesic efficacy of intrathecal morphine and bupivacaine (ITMB) in terms of treating early postoperative pain in adult patients who underwent robotic-assisted laparoscopic prostatectomy (RALP).

**Methods:**

Fifty patients were prospectively enrolled and randomly classified into the non-ITMB (n = 25) and ITMB (n = 25) groups. The ITMB therapeutic regimen consisted of 0.2 mg morphine and 7.5 mg bupivacaine (total 1.7 mL). All patients were routinely administered the intravenous patient-controlled analgesia and appropriately treated with rescue intravenous (IV) opioid drugs, based on the discretion of the attending physicians who were blinded to the group assignments. Cumulative IV opioid consumption and the numeric rating scale (NRS) score were assessed at 1, 6, and 24 h postoperatively, and opioid-related complications were measured during the day after surgery.

**Results:**

Demographic findings were comparable between patients who did and did not receive ITMB. The intraoperative dose of remifentanil was lower in the ITMB group than in the non-ITMB group. Pain scores (i.e., NRS) at rest and during coughing as well as cumulative IV opioid consumption were significantly lower in patients who received ITMB than in those who did not in the post-anesthesia care unit (PACU; i.e., at 1 h after surgery) and the ward (i.e., at 6 and 24 h after surgery). ITMB was significantly associated with postoperative NRS scores of ≤ 3 at rest and during coughing in the PACU (i.e., at 1 h after surgery) before and after adjusting for cumulative IV opioid consumption. In the ward (i.e., at 6 and 24 h after surgery), ITMB was associated with postoperative NRS scores of ≤ 3 at rest and during coughing before adjusting for cumulative IV opioid consumption but not after. No significant differences in complications were observed, such as post-dural puncture headache, respiratory depression, nausea, vomiting, pruritus, or neurologic sequelae, during or after surgery.

**Conclusion:**

A single spinal injection of morphine and bupivacaine provided proper early postoperative analgesia and decreased additional requirements for IV opioids in patients who underwent RALP.

*Trial registration*: Clinical Research Information Service, Republic of Korea; approval number: KCT0004350 on October 17, 2019. https://cris.nih.go.kr/cris/en/search/search_result_st01.jsp?seq=15637

## Background

Robotic-assisted laparoscopic prostatectomy (RALP) is a technically advanced surgical procedure with minimally invasive features that provides a better surgical view and maneuverability than open and/or laparoscopic prostatectomy does [1]. In a large cohort study, RALP seemed to produce a better clinical oncological prognosis, including lower risks of a positive surgical margin, decreased requirement for radiation treatment, and reduced 30-day mortality, compared to open radical prostatectomy [2]. In addition, patients who underwent RALP benefit from favorable functional consequences such as a two-fold higher chance of reaching continence and potency compared to laparoscopic prostatectomy [3]. However, patients who underwent RALP frequently experience considerable pain, particularly during the day after surgery that may result from skin-port incisions, multiple dissections of prostate-involved and surrounding tissues, bladder spasm, and transurethral catheter irritation [4]. Numerous pain control methods, including peripheral and/or central nerve block techniques have been investigated to ameliorate the acute pain that occurs immediately after RALP [5–7].

The analgesic efficacy of intrathecal morphine during open abdominal surgery is better than that of an intravenous (IV) opioid, but the incidence rates of side effects are comparable between the two pain control methods [8]. Another open abdominal surgery study suggested that pain levels assessed using a visual analogue scale at rest and during coughing are more satisfactory after administering intrathecal morphine compared to a ropivacaine wound infusion [9]. Spinal analgesia seems to provide better outcomes in laparoscopy-based minimally invasive surgery than epidural and/or IV analgesia do from the immediate postoperative period until the patient is deemed medically suitable for discharge and during the total hospital stay, in terms of bowel function recovery, pain scores, changes in pulmonary function, and quality of life [10]. A combination of intrathecal morphine and bupivacaine (ITMB) is reported to have additive impacts on early postoperative analgesia in patients undergoing gynecologic surgery [11]. Therefore, ITMB seems to be a potentially acceptable strategy for improving acute pain control after RALP.

The aims of this study were to evaluate pain scores and cumulative IV opioid consumption on day 1 after administering ITMB, and to investigate the association between ITMB and an appropriate analgesic result after adjusting for cumulative IV opioid consumption in patients who underwent RALP. In addition, we compared postoperative complications between patients who did and did not receive ITMB.

## Patients and methods

### Ethical considerations

This single-center, prospective randomized controlled study was approved by the institutional review board of the ethics committee of Seoul St. Mary’s Hospital (approval number: KC19MESI0629 on October 7, 2019) and was performed according to the principles of the Declaration of Helsinki. The study protocol was retrospectively and prospectively registered at a publicly accessible clinical registration site that is recognized by the International Committee of Medical Journal Editors (Clinical Research Information Service, Republic of Korea, approval number: KCT0004350 on October 17, 2019). Written informed consent was obtained from all patients at our hospital who were enrolled between October 2019 and December 2019. Our study adhered to the CONSORT guidelines and includes a completed CONSORT checklist as an Additional file 1.

### Study population

Inclusion criteria for this study were: male gender, age 19–75 years, scheduled for elective RALP for prostate cancer, and American Society of Anesthesiologists (ASA) physical status I or II [12]. Exclusion criteria were: emergency cases; age < 19 or > 75 years; ASA physical status of III–V; intraoperative development of hemodynamic instability and/or massive hemorrhage that required rescue management, such as transfusion of blood products or infusion of a strong vasopressor (i.e., epinephrine or norepinephrine); severe nausea and vomiting that required withholding an additional IV opioid infusion within 1 day after surgery; and refusal to participate in the study.

**Fig. 1:**
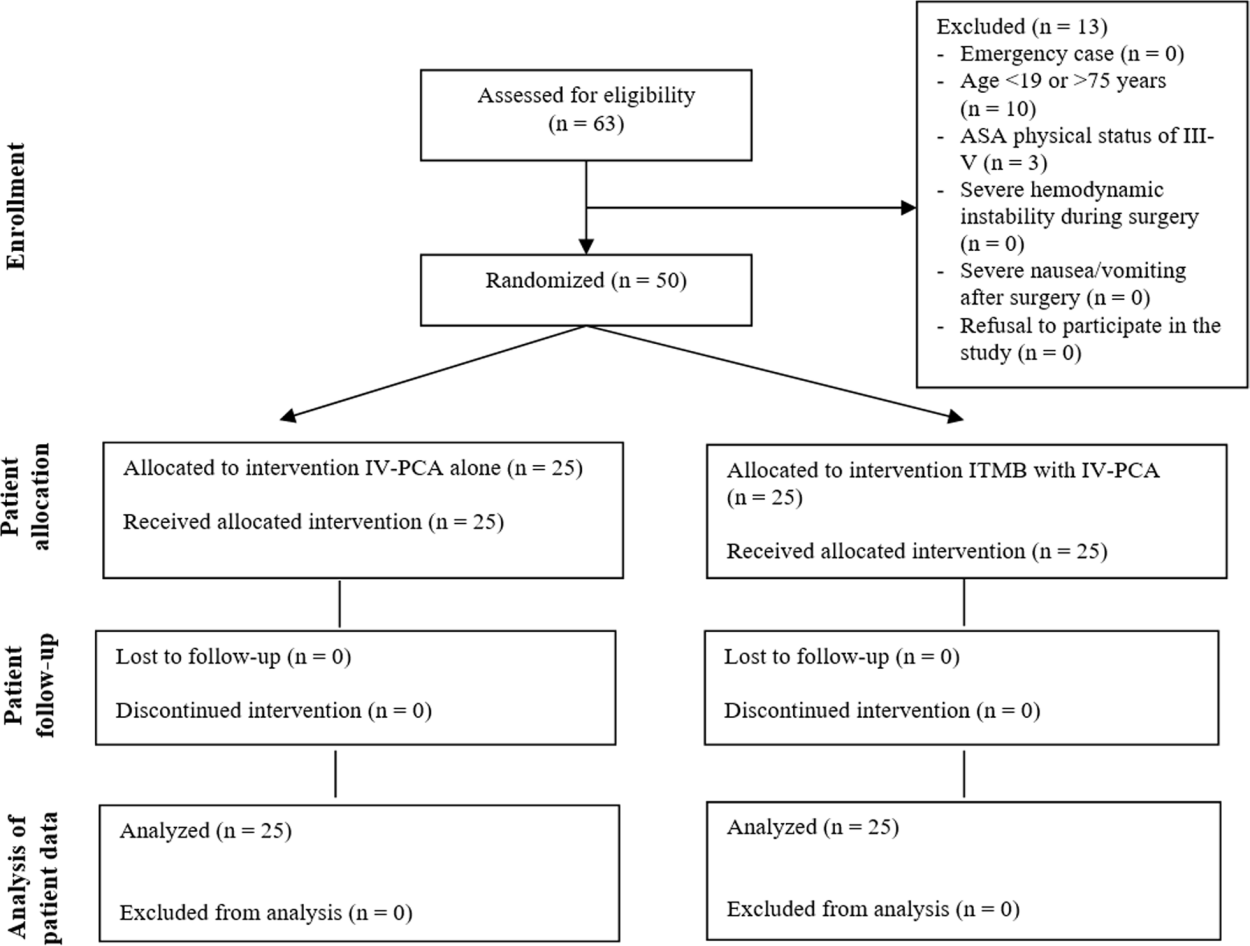
CONSORT diagram showing how study participants went through the different trial phases. English editing service

A total of 63 patients were assessed for eligibility in our study; 13 patients with age > 75 years (n = 10) or ASA physical status of III (n = 3) were excluded, and thus, 50 patients were enrolled in this study (Fig. 1). Randomization was performed using sealed, opaque envelopes. An independent colleague randomly grouped the envelopes in blocks of 10 with a 1:1 ratio to produce an equal distribution across the whole study period. The envelopes were stacked and stored. When an enrolled patient arrived in the holding area, the upper envelope was opened by the attending anesthesiologist. The surgical team, physician, and nurses in the post-anesthesia care unit (PACU) and ward as well as researchers were all blinded to the group allocations. The attending anesthesiologist and nurses in the operating room, who were not involved in further patient care or data collection other than filling in medical record forms, were aware of the group allocations.

### Surgery and anesthesia

RALP was exclusively performed by expert urologists using a robotic-assisted laparoscopic device (Da Vinci Si System; Intuitive Surgical, Sunnyvale, CA, USA). Patients were positioned in the lithotomy position and the operative field was disinfected and draped. CO_2_ gas was insufflated into the abdominal cavity (i.e., pneumoperitoneum) with a pressure of up to 15 mmHg through a 12-mm camera trocar inserted through a periumbilical incision. After inserting the remaining five trocars, including three 8-mm robotic trocars, and 15-mm and 5-mm assisting trocars, intra-abdominal pressure was reduced to 12 mmHg, and the patient was placed in the steep Trendelenburg position at the maximal angle (45°) of the surgical table (Maquet, Rastatt, Baden-Württemberg, Germany); this approach was routinely applied to achieve the optimal surgical view. Intra-abdominal pressure was maintained at 12–15 mmHg during surgery. The surgical position was restored to the supine position and the CO_2_ gas was removed at the time of peritoneal closure.

Balanced anesthesia was provided under standard vital sign monitoring, such as the electrocardiogram, systolic and diastolic blood pressure, heart rate, SpO_2_, body temperature, and capnography, by attending expert anesthesiologists who were aware of the group allocations but were not involved in later patient care or data collection other than the completion of medical records. Propofol (1–2 mg/kg; Fresenius Kabi, Bad Homburg, Germany) and 0.6 mg/kg rocuronium (Merck Sharp and Dohme Corp., Kenilworth, NJ, USA) were infused to induce anesthesia; and 4.0%–6.0% desflurane (Baxter, Deerfield, IL, USA) with medical air/oxygen was provided to maintain anesthesia under a Bispectral Index™ (Medtronic, Minneapolis, MN, USA) of 40–60 to ensure adequate hypnotic depth. Remifentanil (Hanlim Pharm. Co., Ltd., Seoul, Republic of Korea) was continuously infused at a rate of 0.1–0.5 μg/kg/min, as appropriate. Rocuronium was repeatedly administered under train-of-four monitoring (more than one twitch). End-tidal CO_2_ was set between 30 and 40 mmHg by adjusting the mechanical ventilator mode. Hypotensive events were defined as systolic blood pressure < 90 mmHg or diastolic blood pressure < 60 mmHg over 5 min, and treated with rescue IV ephedrine (Daewon Pharm. Co., Ltd., Seoul, Republic of Korea) and/or fluid resuscitation therapy based on the discretion of the attending anesthesiologists, as appropriate.

In the PACU, the attending physicians and nurses were not consulted unless surgical issues, such as massive hemorrhage requiring blood product transfusion, or persistent hemodynamic issues, such as hypotension requiring continuous vasopressor (i.e., epinephrine or norepinephrine) infusion or fluid resuscitation therapy during or after surgery, arose. All patients were transferred to the ward at 1 h after surgery.

### ITMB intervention

None of the patients received sedative premedication in the operating room to allow immediate identification of any nerve injury during the intrathecal intervention performed prior to the induction of general anesthesia. After establishing standard vital sign monitoring, the patient was positioned in the right or left lateral decubitus position, and the skin over the lumbar region was cleaned with chlorhexidine and draped. The patients received 0.2 mg (0.2 mL) of intrathecal morphine sulfate (BCWORLD Pharm. Co., Ltd., Seoul, Republic of Korea) and 7.5 mg (1.5 mL) of bupivacaine (Mitsubishi Tanabe Pharm. Co., Ltd., Osaka, Japan) using a sterile 25G Quincke type-spinal needle (TAE-CHANG Industrial Co., Ltd., Chungcheongnam-do, Republic of Korea) between lumbar vertebrae 3 and 4. Morphine sulfate and bupivacaine (total 1.7 mL) were administered as a single injection after obtaining cerebrospinal fluid.

The entire study population was randomly assigned and classified into the following two groups: patients who did *vs.* those who did not receive ITMB.

### IV opioid administration

All patients undergoing RALP were routinely given IV patient-controlled analgesia (IV-PCA) (AutoMed 3200; Acemedical, Seoul, Republic of Korea), which included 1,000 μg of fentanyl (Dai Han Pharm. Co., Ltd., Seoul, Republic of Korea), 90 mg of ketorolac (Hanmi Pharm. Co., Ltd., Seoul, Republic of Korea), and 0.3 mg of Naseron (Boryung Co., Ltd., Seoul, Republic of Korea). The IV-PCA program consisted of a 2-mL (20 μg of fentanyl) bolus injection and a 0.5-mL (5 μg of fentanyl) basal infusion of the IV-PCA solution with a lockout time of 10 min. The cumulative fentanyl consumption via IV-PCA was assessed at 1, 6, and 24 h after surgery. When patients suffered acute postoperative breakthrough pain (i.e., pain score > 6 on a numeric rating scale [NRS]), pethidine (BCWORLD Pharm. Co., Ltd.) and/or tramadol (YUHAN, Seoul, Republic of Korea) as rescue analgesic IV drugs were administered based on the discretion of the attending physicians in the PACU or the ward, who were blinded to the group assignments. The usage frequencies and doses of the rescue analgesic drugs were recorded. Cumulative IV opioid consumption was calculated from the dose conversion of fentanyl, tramadol, and pethidine into morphine according to the equivalent analgesic dose ratio (i.e., 100 μg of fentanyl = 100 mg of tramadol = 100 mg of pethidine = 10 mg of morphine) [13–15], and assessed at 1, 6, and 24 h after surgery.

### Perioperative findings

The preoperative findings included age, height, weight, body mass index (BMI), comorbidities (i.e., diabetes mellitus [DM] and hypertension), history of abdominal surgery, prostate cancer stage [16], laboratory variables (i.e., prostate-specific antigen, hemoglobin, white blood cell [WBC] count, and neutrophil, lymphocyte, and platelet counts). Intraoperative findings included surgical duration, hypotensive events, rescue IV ephedrine dose, total dose of remifentanil, hourly fluid infusion, hourly urine output, and total blood loss volume. The NRS score (1–3, mild pain; 4–6, moderate pain; 7–10, severe pain) at rest and during coughing was assessed at 1, 6, and 24 h after surgery. In our study, an NRS score ≤ 3 was considered an acceptable pain control target. Development of opioid-related complications on day 1 after surgery was assessed, including post-dural puncture headache, respiratory depression, nausea, vomiting, pruritus, and neurologic sequela.

### Statistical analysis

The minimum sample size was determined based on the difference in cumulative IV opioid consumption on day 1 after surgery between patients who did and did not receive ITMB. Cumulative consumption was calculated from previous electronic medical records at our hospital (unpublished). The mean cumulative IV opioid consumption (morphine equianalgesic dose) on postoperative day 1 by the patients who received no ITMB (n = 10) and ITMB (n = 10) was 45.01 and 23.23 mg, respectively, and the standard deviation (SD) for 20 patients was 25.49 mg. A minimum sample size of 22 patients in each group was required for α = 0.05 and a power of 0.8. Therefore, we recruited 25 patients for each group assuming a dropout rate of 10%.

Values are expressed as the means ± standard deviations, medians with interquartile ranges (IQR) in parentheses, or numbers with percentages in parentheses. The normality of the distribution of continuous data was evaluated using the Shapiro–Wilk test. The perioperative findings were compared between patients who did and did not receive ITMB using the unpaired *t*-test or the Mann–Whitney *U*-test and Pearson’s *χ*^2^ test or Fisher’s exact test, as appropriate. Odds ratios (reported with 95% confidence intervals [CIs] in parentheses) of ITMB with a postoperative NRS score ≤ 3 at rest and during coughing were investigated before and after adjusting for cumulative IV opioid consumption using logistic regression analysis. All tests were two-sided, and a *p*-value < 0.05 was considered significant. Statistical analyses were performed using SPSS for Windows (*ver.* 24.0; IBM Corporation, Armonk, NY, USA) and MedCalc for Windows software (*ver.* 11.0; MedCalc Software, Ostend, Belgium).

## Results

### Demographic findings in patients who underwent RALP

In the present study, all patients (n = 50) were rated as ASA physical status I or II and underwent elective RALP. The median age was 64 (62–68) years, and the median BMI was 24.0 (22.6–26.7) kg/m^2^. Twelve patients (24.0%) had DM and 19 patients (38.0%) had hypertension. Eleven patients (22.0%) were assessed to be at prostate cancer stage I, 32 patients (64.0%) were at stage II, and 7 patients (14.0%) were at stage III. The median prostate specific antigen level was 7.1 (4.9–10.8) ng/mL; the median WBC count, hemoglobin level, and platelet count were 6.2 (5.0–7.4) × 10^9^/L, 14.4 (13.7–15.1) g/dL, and 195.5 (166.3–227.3) × 10^9^/L, respectively.

The median surgical duration was 120 (114–135) min. Twenty-one patients (42.0%) experienced transient hypotensive events, and the median dose of rescue ephedrine was 0 (0–5) mg. There were no cases of persistent hypotension requiring continuous vasopressor (i.e., epinephrine or norepinephrine) infusion or fluid resuscitation therapy during or after surgery. The median dose of total remifentanil was 0.4 (0.2–0.5) mg, and the median levels of crystalloid infusion, urine output, and blood loss were 3.8 (2.9–4.9) mL/kg/h, 0.6 (0.4–0.8) mL/kg/h, and 100 (50–100) mL, respectively.

### Comparison of pre- and intraoperative findings between patients who did or did not receive ITMB

No significant differences were observed in the preoperative findings between patients who did and did not receive ITMB (Table 1). The total remifentanil infusion dose was lower in patients who received ITMB than in those who did not, and the ITMB group exhibited a higher rate of hypotensive events and required a higher rescue ephedrine dose compared to the no-ITMB group, although the differences were not statistically significant. Other intraoperative findings were similar between the two groups (Table 2).

**Table 1:** Comparison of preoperative findings between the patients who did or did not receive intrathecal morphine and bupivacaine (ITMB)

Group	No ITMB	ITMB	*p*
n	25	25	
Age (years)	65.0 (61.5–68.0)	64.0 (62.0–71.5)	0.853
Height (cm)	170.0 (166.3–172.0)	169.0 (164.1–172.5)	0.69
Weight (kg)	68.0 (61.5–78.0)	67.0 (62.5–75.5)	0.593
Body mass index (kg/m^2^)	23.9 (22.7–27.2)	24.1 (22.2–26.5)	0.438
*Comorbidity*			
Hypertension	7 (28.0%)	12 (48.0%)	0.145
Diabetes mellitus	7 (28.0%)	5 (20.0%)	0.508
History of abdominal surgery	6 (24.0%)	5 (20.0%)	0.733
Prostate cancer stage			0.482
Stage I	4 (16.0%)	7 (28.0%)	
Stage II	18 (72.0%)	14 (56.0%)	
Stage III	3 (12.0%)	4 (16.0%)	
*Laboratory variables*			
Prostate-specific antigen (ng/mL)	7.0 (4.5–12.4)	7.4 (5.1–10.5)	0.954
Hemoglobin (g/dL)	14.4 (14.0–15.0)	14.5 (13.4–15.3)	0.727
White blood cell count (× 10^9^/L)	5.5 (4.7–7.1)	6.9 (5.2–7.8)	0.162
Neutrophils (%)	54.9 (50.7–58.1)	54.8 (51.4–55.0)	0.362
Lymphocytes (%)	33.6 (30.3–36.2)	34.0 (33.5–38.8)	0.299
Platelet count (× 10^9^/L)	188.0 (162.5–219.0)	202.0 (170.5–233.5)	0.26

Values are expressed as the mean ± standard deviation, medians with interquartile ranges in parentheses, or numbers with percentages in parentheses

**Table 2:** Comparison of intraoperative findings between patients who did or did not receive ITMB

Group	No ITMB	ITMB	*p*
n	25	25	
Surgical duration (min)	120 (108–143)	120 (115–130)	1.000
Hypotensive event^a^	8 (32.0%)	13 (52.0%)	0.152
Rescue ephedrine dose (mg)	0.0 (0.0–4.0)	4.0 (0.0–8.0)	0.095
Remifentanil dose (mg)	0.5 (0.4–0.6)	0.2 (0.2–0.3)	< 0.001
Hourly fluid infusion (mL/kg/h)	3.5 (3.0–4.2)	4.3 (2.8–6.0)	0.233
Hourly urine output (mL/kg/h)	0.6 (0.4–0.7)	0.7 (0.4–0.9)	0.322
Blood loss (mL)	100 (50–100)	100 (50–150)	0.449

Values are expressed as the mean ± standard deviation, medians with interquartile ranges in parentheses, or numbers with percentages in parentheses

^a^A hypotensive event was defined as systolic blood pressure < 90 mmHg or diastolic blood pressure < 60 mmHg over 5 min

### Analysis of postoperative NRS score and cumulative IV opioid consumption between patients who did and did not receive ITMB

The NRS score and cumulative IV opioid consumption after surgery were compared between the two groups (Table 3). In the PACU (i.e., at 1 h after surgery), incidences of mild pain (i.e., NRS score ≤ 3) at rest and during coughing were higher in the ITMB group (68.0% at rest and 52.0% during coughing) than in the no-ITMB group (12.0% at rest and 4.0% during coughing). In the ward (i.e., at 6 h after surgery), the incidences of mild pain at rest and during coughing were higher in the ITMB group (84.0% at rest and 52.0% during coughing) than in the no-ITMB group (20.0% at rest and 12.0% during coughing). In the ward (i.e., at 24 h after surgery), the incidences of mild pain at rest and during coughing were higher in the ITMB group (92.0% at rest and 52.0% during coughing) than in the no-ITMB group (68.0% at rest and 16.0% during coughing). The median (IQR) cumulative level of IV opioid consumption was significantly lower in the ITMB group than in the no-ITMB group—4.4 (2.9–5.7) versus 12.1 (5.8–14.0) mg, respectively, in the PACU; 8.4 (6.0–10.2) versus 18.5 (12.7–28.4) mg, respectively, in the ward (i.e., at 6 h after surgery); and 18.7 (15.0–24.2) versus 38.4 (26.6–57.1) mg, respectively, in the ward (i.e., at 24 h after surgery).

**Table 3:** Comparison of postoperative numeric rating scale (NRS) score and cumulative intravenous (IV) opioid consumption between patients with/without ITMB

Group	No ITMB	ITMB	*p*
n	25	25	
**At 1 h after surgery (in the PACU)**			
*NRS score at rest*			< 0.001
1–3	3 (12.0%)	17 (68.0%)	
4–6	13 (52.0%)	5 (20.0%)	
7–10	9 (36.0%)	3 (12.0%)	
*NRS score during coughing*			< 0.001
1–3	1 (4.0%)	13 (52.0%)	
4–6	8 (32.0%)	7 (28.0%)	
7–10	16 (64.0%)	5 (20.0%)	
Cumulative IV opioid consumption (mg)^a^	12.1 (5.8–14.0)	4.4 (2.9–5.7)	0.001
**At 6 h after surgery (in the ward)**			
*NRS score at rest*			< 0.001
1–3	5 (20.0%)	21 (84.0%)	
4–6	13 (52.0%)	3 (12.0%)	
7–10	7 (28.0%)	1 (4.0%)	
*NRS score during coughing*			0.009
1–3	3 (12.0%)	13 (52.0%)	
4–6	13 (52.0%)	8 (32.0%)	
7–10	9 (36.0%)	4 (16.0%)	
Cumulative IV opioid consumption (mg)^a^	18.5 (12.7–28.4)	8.4 (6.0–10.2)	< 0.001
**At 24 h after surgery (in the ward)**			
*NRS score at rest*			0.034
1–3	17 (68.0%)	23 (92.0%)	
4–6	8 (32.0%)	2 (8.0%)	
7–10	0 (0.0%)	0 (0.0%)	
*NRS score during coughing*			0.024
1–3	4 (16.0%)	13 (52.0%)	
4–6	18 (72.0%)	11 (44.0%)	
7–10	3 (12.0%)	1 (4.0%)	
Cumulative IV opioid consumption (mg)^a^	38.4 (26.6–57.1)	18.7 (15.0–24.2)	< 0.001

Values are expressed as medians with interquartile ranges in parentheses, or numbers with percentages in parentheses

*PACU* post-anesthesia care unit

^a^Morphine equianalgesic dose (mg)

We also assessed whether there was an association between ITMB and postoperative NRS score ≤ 3 at rest and during coughing in the PACU and ward (Table 4). In the PACU (i.e., at 1 h after surgery), ITMB was significantly associated with postoperative NRS scores ≤ 3 at rest both before (*β* = 2.746; odds ratio = 15.583; 95% CI  3.583–67.784; *p* < 0.001) and after adjusting for cumulative IV opioid consumption (*β* = 2.092; odds ratio = 8.098; 95% CI  1.258–52.133; *p* = 0.028). ITMB was significantly associated with postoperative NRS scores ≤ 3 during coughing both before (*β* = 3.258; odds ratio = 26.0; 95% CI  3.032–222.928; *p* = 0.003) and after adjusting for cumulative IV opioid consumption (*β* = 2.61; odds ratio = 13.593; 95% CI  1.344–137.478; *p* = 0.027). In the ward (i.e., at 6 h after surgery), ITMB was significantly associated with postoperative NRS score ≤ 3 at rest before (*β* = 3.045; odds ratio = 21.0; 95% CI  4.924–89.561; *p* < 0.001), but not after adjusting for cumulative IV opioid consumption. ITMB was significantly associated with postoperative NRS score ≤ 3 during coughing before (*β* = 2.072; odds ratio = 7.944; 95% CI  1.884–33.498; *p* = 0.005), but not after adjusting for cumulative IV opioid consumption. In the ward (i.e., at 24 h after surgery), ITMB was significantly associated with postoperative NRS score ≤ 3 at rest before (*β* = 1.689; odds ratio = 5.412; 95% CI  1.017–28.791; *p* = 0.048), but not after adjusting for cumulative IV opioid consumption. ITMB was significantly associated with postoperative NRS score ≤ 3 during coughing before (*β* = 1.738; odds ratio = 5.687; 95% CI  1.51–21.424; *p* = 0.01), but not after adjusting for cumulative IV opioid consumption.

**Table 4:** Association between ITMB and postoperative NRS score ≤ 3 at rest and during coughing in the PACU and ward

	*β*	Odds ratio	95% CI	*p*
**At 1 h after surgery (in the PACU)**				
*NRS score* ≤ *3 at rest*				
IV-PCA alone	Reference			
ITMB and IV-PCA	2.746	15.583	3.583–67.784	< 0.001
ITMB and IV-PCA^a^	2.092	8.098	1.258–52.133	0.028
*NRS score* ≤ *3 during coughing*				
IV-PCA alone	Reference			
ITMB and IV-PCA	3.258	26.0	3.032–222.928	0.003
ITMB and IV-PCA^a^	2.610	13.593	1.344–137.478	0.027
**At 6 h after surgery (in the ward)**				
*NRS score* ≤ *3 at rest*				
IV-PCA alone	Reference			
ITMB and IV-PCA	3.045	21.0	4.924–89.561	< 0.001
ITMB and IV-PCA^a^	1.713	5.547	0.804–38.249	0.082
*NRS score* ≤ *3 during coughing*				
IV-PCA alone	Reference			
ITMB and IV-PCA	2.072	7.944	1.884–33.498	0.005
ITMB and IV-PCA^a^	− 0.030	0.971	0.133–7.079	0.976
**At 24 h after surgery (in the ward)**				
*NRS score* ≤ *3 at rest*				
IV-PCA alone	Reference			
ITMB and IV-PCA	1.689	5.412	1.017–28.791	0.048
ITMB and IV-PCA^a^	0.572	1.771	0.219–14.329	0.592
*NRS score ≤ 3 during coughing*				
IV-PCA alone	Reference			
ITMB and IV-PCA	1.738	5.687	1.51–21.424	0.01
ITMB and IV-PCA^a^	0.776	2.172	0.439–10.745	0.342

*IV-PCA* intravenous patient-controlled analgesia, *CI* confidence interval

^a^After adjustment for cumulative IV opioid consumption

### Comparison of postoperative complications between patients who did or did not receive ITMB

No significant differences in complications, such as post-dural puncture headache, respiratory depression, nausea, vomiting, pruritus, or neurologic sequelae, were detected on day 1 after surgery between the two patient groups (Table 5).

**Table 5:** Comparison of complications on day 1 after surgery between patients who did or did not receive ITMB

Group	No ITMB	ITMB	*p*
n	25	25	
Post-dural puncture headache	0 (0.0%)	0 (0.0%)	1.000
Respiratory depression	0 (0.0%)	0 (0.0%)	1.000
Nausea	2 (8.0%)	6 (24.0%)	0.247
Vomiting	0 (0.0%)	1 (4.0%)	1.000
Pruritus	0 (0.0%)	4 (16.0%)	0.110
Neurologic sequela	0 (0.0%)	0 (0.0%)	1.000

Values are expressed as numbers with percentages in parentheses

## Discussion

The main findings of our study are that a low dose of intrathecal morphine (i.e., 0.2 mg) and 0.5% bupivacaine (i.e., 7.5 mg) had a significant analgesic benefit during the first 24 h postoperatively in patients who underwent RALP. Patients who received ITMB reported lower pain levels and required less cumulative IV opioids than those who did not receive ITMB. In the PACU, ITMB seemed to play a predominant role in achieving an optimal analgesic level (i.e., NRS score ≤ 3) at rest and during coughing, independent of cumulative IV opioid consumption. In the ward, ITMB was associated with attenuating pain level. In addition, the incidence rates of nausea/vomiting and pruritus were marginally higher in patients who received ITMB than in those who did not, but no post-dural puncture headache, respiratory depression, or neurologic sequelae occurred in the patients who received ITMB.

RALP is a minimally invasive surgery requiring small incisions; however, patients frequently suffer moderate to severe pain for the first 24 h postoperatively due to visceral irritation and a prolonged pneumoperitoneum with a high CO_2_ pressure. Thereafter, patients experience a gradual decrease in pain severity [17, 18]. The intrathecal approach is safe and acceptable for acute postoperative pain control and has several advantages over systemic and/or epidural pain-relief methods, including less invasiveness, a smaller opioid requirement for providing a similar analgesic effect, and a lower risk of procedural failure [5, 10, 19]. Due to the hydrophilic nature of morphine, the analgesic effect of single-shot intrathecal morphine continuously maintained for 24 h after surgery may be suitable for treating pain due to laparoscopic surgery [20]. As part of multimodal pain management, single-shot intrathecal morphine contributes to optimal pain recovery after minimally invasive surgery [21, 22]. Bae et al*.*[19] reported that the pain score (i.e., numerical pain score at rest and during coughing) was lower and less morphine was required during the first 24 h after RALP in patients who received 300 μg of intrathecal morphine than in those who did not receive intrathecal morphine. The incidence rates of opioid-related complications (i.e., nausea, vomiting, dizziness, headache, and pruritus) were comparable between patients who did and did not receive intrathecal morphine.

A higher morphine dose (e.g., > 500 μg) ensures a strong analgesic effect, but the probability of complications such as nausea, vomiting, and respiratory depression increases in line with the morphine dose [23, 24]. A relatively low dose of morphine (e.g., 100–400 μg) with a local anesthetic (e.g., bupivacaine) may allow the appropriate analgesic target to be reached without side effects in patients undergoing laparoscopic surgery [5, 25, 26]. Nguyen et al. [26] suggested that a regimen of 0.4 mg of intrathecal morphine and 15 μg of fentanyl with an additional 15 mg of 0.5% bupivacaine significantly decreases IV morphine consumption rate and the pain score compared to using intrathecal opioids alone after laparoscopic liver surgery. An intrathecal mixture of morphine (200 μg in patients ≤ 75 years or 150 μg in patients > 75 years) and 10 mg 0.5% bupivacaine was associated with a lower dose of opioid infusion in patients in the enhanced recovery after surgery program for laparoscopic colonic resection [25]. Sherif et al. [22] reported that the analgesic impact (i.e., visual analogue scale score and total morphine consumption) and recovery outcomes (i.e., time to first ambulation, return of intestinal sounds, and hospital stay) were superior in patients who received 0.3 mL (0.3 mg) of intrathecal morphine and 1.2 mL of 0.5% bupivacaine than in patients who received 0.3 mL of intrathecal saline and 1.2 mL of 0.5% bupivacaine after laparoscopic bariatric surgery. Patients who underwent RALP and received 300 μg of intrathecal morphine and 12.5 mg of bupivacaine exhibited better early postoperative recovery, as represented by the Quality of Recovery-15 questionnaire score on postoperative day 1, than those who received a subcutaneous sham injection or 0.1-mg/kg load of IV morphine loading [5]. The optimal spinal dose of bupivacaine for the recovery of motor function and guaranteed hospital discharge in patients undergoing ambulatory surgery is 7.5 mg; this dose was able to resolve motor block within 5 h and achieve discharge within 6 h in 95% of patients [27]. Our study suggested that an intrathecal low-dose regimen (i.e., 0.2 mg of morphine and 7.5 mg of 0.5% bupivacaine) may be effective for acute pain control in the PACU (i.e., at 1 h after surgery) and the ward (i.e., at 6 and 24 h after surgery) without fatal complications. In particular, this regimen may be able to provide a significantly better analgesic effect in the PACU patients (i.e., eightfold higher score at rest and 14-fold higher score during coughing after adjusting for IV opioid dose) compared to outcomes from using IV-PCA alone. This regimen did not significantly affect intraoperative hemodynamic factors (i.e., hypotensive events or the requirement for a rescue dose of ephedrine) or the incidence of postoperative complications (i.e., post-dural puncture headache, respiratory depression, and neurological deficit). However, as intrathecal bupivacaine-induced hemodynamic disturbance, such as hypotension, may be aggravated in vulnerable patients, such as those of advanced age, due to hypovolemia or decreased sympathetic tone and baroreceptor activity, it is necessary to meticulously adjust the dose of additive bupivacaine while taking age into consideration [28, 29].

## Limitations

This study had some limitations that should be discussed. First, our patients were treated for postoperative pain using numerous IV opioids, including fentanyl in IV-PCA, and pethidine and/or tramadol as IV rescue analgesic drugs. Although cumulative IV opioid consumption was calculated by dose conversion of fentanyl, tramadol, and pethidine into morphine according to the equivalent analgesic dose ratio, these calculated doses may be less precise compared to directly measuring the actual amounts of the IV opioid drugs. Second, we were unable to blind the patients and the attending anesthesiologists to the group assignments. The intrathecal practice was performed before inducing anesthesia to avoid potential neurological complications such as nerve injury. However, there were no patients with surgical or hemodynamic issues required consultation from the attending physicians and nurses in the PACU and ward (who recorded the study data), and therefore, the latter were blinded to the group assignments. Third, the sample size was calculated to allow detection of a difference in cumulative IV opioid consumption on day 1 after surgery between patients who did and did not receive ITMB. However, it might not have been sufficient to compare the groups with regard to differences in postoperative complications, such as the rate of intraoperative hypotensive events. Further studies with larger populations are required to elucidate the association between ITMB and postoperative complications. Fourth, although our regimen, which used a lower dose compared to a previous RALP study [5], led to acceptable analgesic benefits during the first 24 h after surgery, we are unable to determine the optimal doses of ITMB for RALP. As the analgesic mechanisms differ between morphine and bupivacaine [30, 31], further studies are required to investigate the dose–response relationship and synergistic effects between these two analgesic drugs.

## Conclusion

A single spinal injection of morphine and bupivacaine provided proper early postoperative analgesia and decreased additional requirements for IV opioids in patients who underwent RALP. The patients who received ITMB were intraoperatively stable and tolerated postoperative spinal practice-related complications. ITMB is considered a critical analgesic modality as part of a multimodal analgesia recovery strategy for RALP.

## Supplementary Information

**Additional file 1.** CONSORT 2010 checklist of information to include when reporting a randomised trial.

## Data Availability

The datasets used and/or analyzed during this study are available from the corresponding author on reasonable request.

## Abbreviations

RALP: Robotic-assisted laparoscopic prostatectomy
IV: Intravenous
ITMB: Intrathecal morphine and bupivacaine
ASA: American Society of Anesthesiologists
PACU: Post-anesthesia care unit
IV-PCA: Intravenous patient-control analgesia
NRS: Numeric rating scale
DM: Diabetes mellitus
WBC: White blood cell
SD: Standard deviation
IQR: Interquartile
